# Solubilization Mechanism of Eco-Friendly Fluorocarbon Surfactants for Fluorinated Monomers: Perfluorinated Chain Length Effect and Dynamic Thermal Response

**DOI:** 10.3390/polym18172056

**Published:** 2026-08-24

**Authors:** Yanrong Chen, Yonghua Shang, Kai Wang, Linjie Wang, Xiaolai Zhang

**Affiliations:** 1 Key Laboratory of Colloid and Interface Chemistry of Ministry of Education, School of Chemistry and Chemical Engineering, Shandong University, Jinan 250100, China; 2 Yantai Economic & Technological Development Area, Wanhua Chemical Group Co., Ltd., No.3 Sanya Road, Yantai 264006, China; 3 Shandong Dongyue Future Hydrogen Energy Materials Co., Ltd., Dongyue Economic Development Zone, Zibo 256401, China; 4 Shandong Key Laboratory of Green Electricity & Hydrogen Science and Technology, School of Chemical Engineering, Shandong Institute of Petroleum and Chemical Technology, Dongying 257061, China

**Keywords:** MD simulation, fluorinated monomers, eco-friendly zwitterionic fluorocarbon surfactant, solubilization mechanism

## Abstract

Extremely hydrophobic fluoromonomers are highly prone to phase separation during emulsion polymerization. Overcoming macroscopic experimental limitations, this study employs all-atom molecular dynamics (AA-MD) simulations to investigate the molecular-level solubilization behavior and thermal adaptability of the novel eco-friendly zwitterionic fluorocarbon surfactant-perfluorohexyl (butyl) sulfonyl carboxy propylamino dimethyl betaine (PFSC) and the traditional hydrocarbon surfactant SDS toward the monomers tetrafluoroethylene (TFE) and perfluoromethyl vinyl ether (PMVE). Simulation results indicate that fluorinated monomers in the SDS system exhibit a more dispersed spatial distribution, with weaker local association in surfactant-enriched regions. In contrast, fluorinated monomers in the PFSC system tend to distribute within regions rich in perfluorinated segments. This spatial characteristic is consistent with thermodynamic analysis results dominated by solubility parameter matching and van der Waals interactions. Under high-temperature conditions, the perfluorohexyl sulfonyl carboxy propylamino dimethyl betaine (C6) system maintains relatively stable local spatial characteristics, with the fluorinated monomers exhibiting low migration behavior. These spatial distribution characteristics and thermal response behaviors suggest that a fluorine-rich environment may help preserve the local distribution of fluorinated monomers under high-temperature conditions. These findings provide molecular-level insights into the structure–property relationships of eco-friendly fluorinated surfactants and offer computational guidance for their rational design.

## 1. Introduction

Benefiting from excellent thermodynamic stability [1,2,3] and extremely low interfacial free energy [4,5,6], fluoropolymers play a significant role in various industrial fields, including semiconductor manufacturing [7,8,9,10], aerospace [2,11,12,13,14], electronic equipment protection [2,5,9], medical devices [6], and surface treatment [4,6,10]. TFE and PMVE, as core monomers for synthesizing high-performance fluoroelastomers [9,15,16], possess a fully fluorinated molecular backbone and extremely high C-F bond density compared to partially fluorinated systems [17]. While this highly fluorinated intrinsic structure imparts excellent physicochemical resistance to the molded polymer network, it also results in low polarizability of the monomer molecules and poor thermodynamic affinity in aqueous media [18]. In industrial emulsion polymerization systems, the solubilization efficiency of surfactants for monomers directly governs the macroscopic polymerization kinetics [19,20]. However, the intense hydrophobic repulsion and significant interphase mass transfer resistance of perfluorinated monomers severely limit effective solubilization within the system. This not only highlights the scientific necessity of elucidating microscopic solubilization mechanisms but also poses rigorous challenges for the molecular engineering design and interfacial regulation of surfactants.

To effectively overcome the intense hydrophobic repulsion of perfluorinated monomers, surfactants must possess both exceptional interfacial activity and affinity for the fluorinated phase [21]. Although the traditional high-efficiency emulsifier perfluorooctanoic acid (PFOA) possesses excellent emulsifying and solubilizing properties, it is subject to increasingly stringent regulatory restrictions due to its environmental persistence, bioaccumulation, and potential toxicological risks [22,23,24], environmentally friendly alternatives such as surfactant compounding [25,26] or the development of reactive emulsifiers [27] have emerged. However, when dealing with extremely hydrophobic systems, these conventional strategies are often hindered by a sharp decline in intrinsic emulsification efficiency or adverse interference with macroscopic polymerization kinetics. To achieve a balance between reducing environmental risks and ensuring emulsification efficiency, the development of new short-carbon-chain fluorinated surfactants has become the mainstream trend for the green transition in this field [28]. However, while shortening the perfluorinated carbon chain effectively reduces environmental persistence and toxicity, it inevitably weakens the intrinsic hydrophobic driving force of the molecules [29], leading to decreased surface activity and impaired solubilization capacity for perfluorinated monomers. Therefore, establishing a reasonable balance between environmental friendliness, interfacial activity, monomer compatibility, and stability during the polymerization process remains a key challenge in the molecular design and synthesis of eco-friendly fluorocarbon surfactants. To break through this performance bottleneck, Zhang et al. recently designed and synthesized new eco-friendly zwitterionic betaine fluorocarbon surfactants (C6 and C4) based on perfluorohexyl and perfluorobutyl groups. Preliminary experiments showed that this series of surfactants exhibits excellent surface activity; C6 can reduce the surface tension of water to 12 mN/m and demonstrated good emulsifying capacity in the emulsion polymerization of methyl methacrylate and dodecafluoroheptyl methacrylate [30]. This set of surfactants with a clear chain-length gradient provides a suitable structural reference model for investigating the effect of fluorocarbon chain length on the solubilization mechanism.

However, the applicability of this new surfactant in TFE/PMVE copolymerization systems has not yet been reported. Whether the short fluorocarbon chains in their structure can effectively capture and stabilize these highly volatile molecules at the atomic scale remains to be elucidated [31,32]. How the differences in perfluorinated chain length affect the monomer capture efficiency of the system for fluorinated monomers at the atomic scale, the nature of the energy driving force for the solubilization structure, specifically the competition between van der Waals and electrostatic forces, and the impact of polymerization temperature on the stability of the solubilization microdomain are details that remain difficult to observe directly through experimental means [33].

To overcome the limitations of experimental observation and fill the theoretical gaps in the system’s mechanism, it is particularly necessary to employ molecular dynamics simulations to reveal interfacial assembly behavior at the atomic scale [33,34]. In view of this, this study selects two novel surfactants, C4 and C6 [30], to construct an aqueous solubilization microenvironment with TFE/PMVE monomers. Simultaneously, the typical hydrocarbon surfactant SDS is introduced as a baseline control system, aiming to intuitively reveal the intrinsic influence of molecular structural characteristics on the solubilization efficiency of extremely hydrophobic monomers through significant differences in physicochemical properties.

In this study, AA-MD simulations were employed to systematically evaluate the potential solubilization mechanisms of various systems [35,36]. By constructing all-atom simulation systems with different perfluorinated chain lengths and temperature gradients, the compatibility between surfactants and monomers was first quantitatively assessed using solubility parameters as an indicator [34,35]. Subsequently, a multi-level analysis of the underlying solubilization mechanism was conducted across several dimensions, including microscopic configurations [36], relative concentration distributions [36,37,38], non-bonded interaction energy decomposition [34], and monomer capture efficiency [34,35,38]. Furthermore, the dynamic structural evolution and stability of each system under different thermodynamic fields were revealed by combining the radius of gyration [39] and molecular diffusion behavior [34]. This research aims to fill the mechanistic gap regarding such amphoteric surfactants in the field of fluorinated monomer solubilization, and is expected to provide key theoretical support for the microscopic mechanism verification and targeted optimization of fluorinated emulsion systems under polymerization conditions.

## 2. Simulation Methods

### 2.1. Simulation Software and Force Field Selection

All molecular dynamics simulations in this study were performed using the BIOVIA Materials Studio (MS) 2020 software package, with the COMPASS II force field selected for all energy calculations. This force field utilizes parameters developed based on ab initio calculations, enabling the accurate prediction of the structure, conformation, and thermodynamic properties of multi-component complex systems, including gas phases, liquid phases, and polymers [40,41].

### 2.2. Molecular Modeling and Initial Configuration Optimization

**Figure 1:**
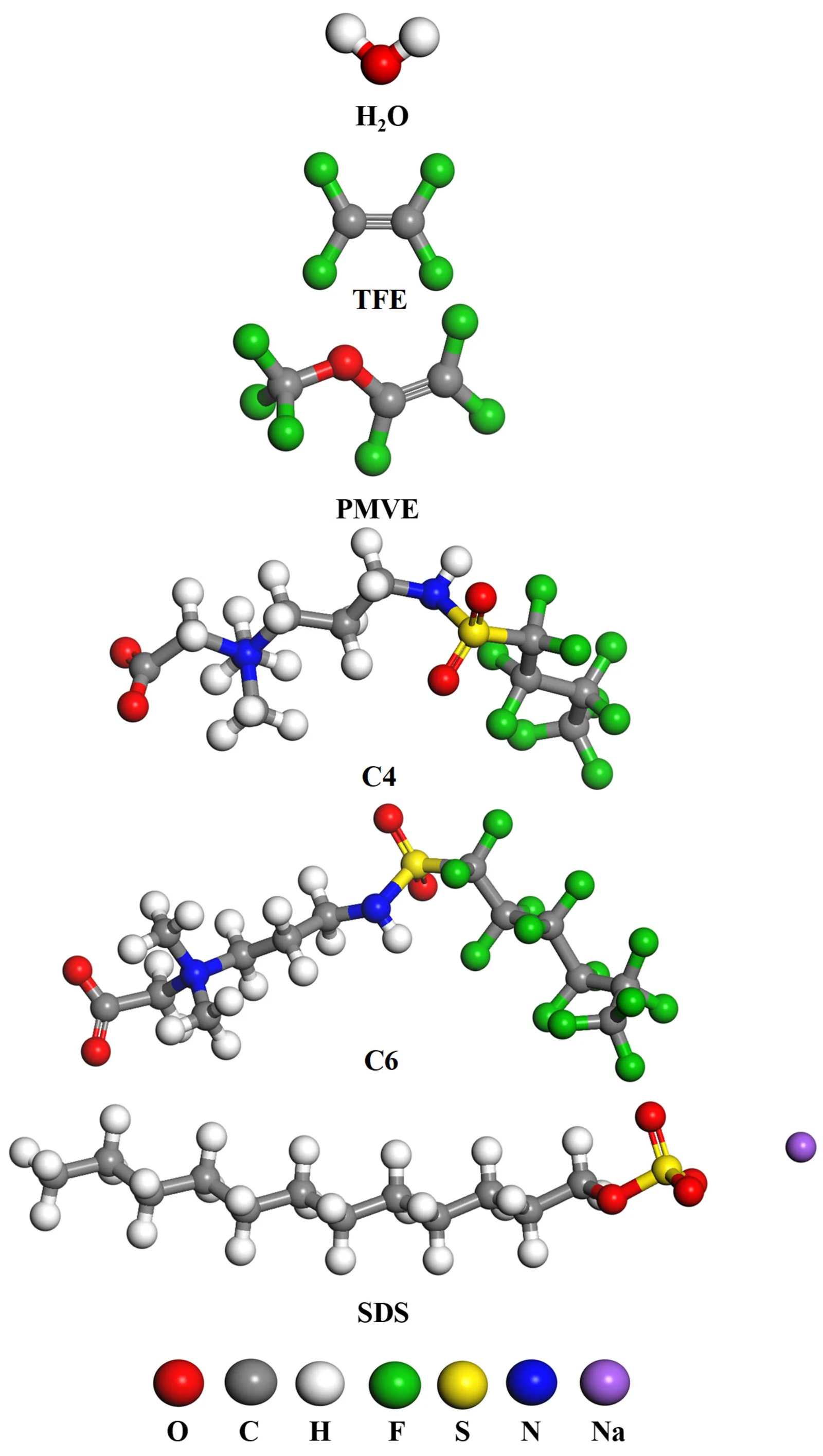
Molecular structural models and atomic color codes of simulated systems.

The structures of TFE and PMVE monomers, as well as SDS, C4, and C6 surfactant molecules, were constructed using the Visualizer Tools module (Figure 1). To ensure the global minimum energy configuration of the molecules was obtained, geometry optimization tasks based on the SMART algorithm were performed on each individual molecule, combined with an annealing process for structural relaxation.

### 2.3. Construction of the Simulation System

The Amorphous Cell (AC) module was utilized to construct periodic three-dimensional simulation cells containing water, monomers, and various surfactants. The solubilization simulation systems are listed in Table 1. To avoid local high-energy conformations caused by random packing, 10 initial configurations were generated for each system. The configuration with the lowest energy was then selected as the final starting point for further geometry optimization.

### 2.4. Simulation Parameters and Thermal Equilibration Process

The time step for all MD simulations was uniformly set to 1 fs. For non-bonded interactions, the electrostatic potential was described using the Ewald summation method, while van der Waals forces were calculated using an atom-based summation method with a cutoff radius of 12.5 Å. The molecular dynamics equilibration and production simulations of the system were conducted in three sequential stages. First, a 200 ps NVT pre-equilibration was performed at the target temperature (298.15 K, 318.15 K, or 338.15 K) to eliminate unreasonable spatial overlaps within the initial conformation and allow for preliminary relaxation of molecular positions. Subsequently, the system entered a 500 ps NPT stabilization phase, during which a Berendsen barostat was employed to adaptively scale the simulation box volume until the overall density and energy of the system converged to the equilibrium state at the corresponding temperature. Finally, the average equilibrium volume from the NPT phase was extracted, and the system size was fixed to perform an 800 ps NVT production simulation. The temperature during this process was precisely maintained by a Nosé thermostat, and all subsequent conformational data used for analysis were extracted from the trajectories of this production stage.

## 3. Results and Discussion

### 3.1. Validation of Simulation Force Field and Parameters: Surface Tension Analysis

Before conducting an in-depth study of the microscopic solubilization mechanism, to ensure the accuracy of the selected COMPASS II force field and the constructed PFSC (C4 and C6) molecular models, this study first calculated their surface tension in a gas–liquid interface system and cross-validated the results with existing macroscopic experimental data. The interfacial tension of the system was calculated according to the Kirkwood–Buff formula [34]:

(1) $$\gamma \;=\frac{1}{2}L_{z}\left(P_{\mathrm{zz}}-\frac{P_{\mathrm{xx}}+P_{\mathrm{yy}}}{2}\right)$$

where *γ* is the surface tension in mN/m; *P_xx_*, *P_yy_*, and *P_zz_* are the components of the system pressure tensor along each axis in 0.1 MPa; and *L_z_* is the length of the simulation box along the *Z*-axis perpendicular to the gas–liquid interface in nm.

The surface tensions of the gas–liquid interfaces for different surfactant systems were simulated, and the results are shown in Table 2. The simulated interfacial tension for the C6 system is 10.37 mN/m, while the experimentally measured interfacial tension in the literature is 12 mN/m [30], representing an error of −13.58%. The simulated interfacial tension for the C4 system is 13.62 mN/m, while the experimentally measured interfacial tension in the literature is 15 mN/m [30], representing an error of −9.2%. As can be seen from the results, the errors for both models are within 15%, and the molecular simulation results are in good agreement with the experimental values. Therefore, these results suggest that the simulation methods and details used in this work are reliable, and the force field models and simulation methods adopted can provide a reasonable description of the interfacial properties of the surfactant molecules. Furthermore, the C6 system exhibits a lower interfacial tension than the C4 system, indicating that the longer the perfluorinated segment, the stronger hydrophobicity and oleophobicity drive the molecules into a more tightly oriented arrangement at the interface, thereby demonstrating a greater ability to reduce interfacial free energy.

### 3.2. Thermodynamic Compatibility Prediction of Surfactants and Monomers: Cohesive Energy Density and Solubility Parameters

Under conditions of constant temperature and pressure, when the surfactant dissolves into the TFE and PMVE mixed copolymerization system, the change in the system’s Gibbs free energy is negative and can be described by Equation (2) [35]:

Δ*G*_m_ = Δ*H*_m_ − *T*Δ*S*_m_ < 0 (2)

where Δ*G*_m_ is the molar Gibbs free energy change in the system, and Δ*H*_m_ is the molar enthalpy change; *T* is the thermodynamic temperature; and Δ*S*_m_ is the molar entropy change. During the process of the surfactant dissolving into the TFE/PMVE system, the surfactant molecules and TFE/PMVE molecules transition from an ordered state to a disordered state, resulting in a system entropy change greater than zero. Therefore, the smaller the molar enthalpy change in the system, the more significant the dissolution tendency of the surfactant in TFE/PMVE. The Hildebrand solubility parameter can be expressed by Equation (3) [42]:

(3) $$\Delta H_{m}\;=\;V_{m}\phi_{1}\phi_{2}(\sqrt{e_{\mathrm{co}h1}}\;-\sqrt{e_{\mathrm{co}h2}}{)}^{2}$$

where *V_m_* is the molar volume of mixing; ϕ_1_ and ϕ_2_ represent the volume fractions of the surfactant and the monomer, respectively; and *e_coh1_* and *e_coh2_* correspond to their respective cohesive energy densities. Since the solubility parameter *δ* is defined as the square root of the cohesive energy density (CED) [43], Equation (3) can also be transformed into Equation (4):

(4) $$\Delta H_{m}\;=\;V_{m}\phi_{1}\phi_{2}(\delta_{1\;}-\delta_{2}{)}^{2}$$

In the formula, *δ*_1_ and *δ*_2_ represent the solubility parameters of the surfactant and the fluorinated monomer, respectively. As core physical quantities for measuring the solubility of molecules in a specific solvent, the difference between their solubility parameters approaches zero when the cohesive energy densities of the two phases are similar, leading to a significant reduction in the enthalpy of mixing. Driven by the effect of increasing entropy, the Gibbs free energy of the system is more likely to become negative, thereby promoting miscibility and the formation of stable solubilized microdomains. This principle constitutes the quantitative theoretical foundation of the “like dissolves like” empirical rule. Therefore, from a thermodynamic perspective, a smaller difference in the solubility parameters between the surfactant and the monomer indicates better mutual compatibility, which can reduce the interfacial free-energy penalty and favor the partitioning and accumulation of monomers within the surfactant-rich domain.

**Figure 2:**
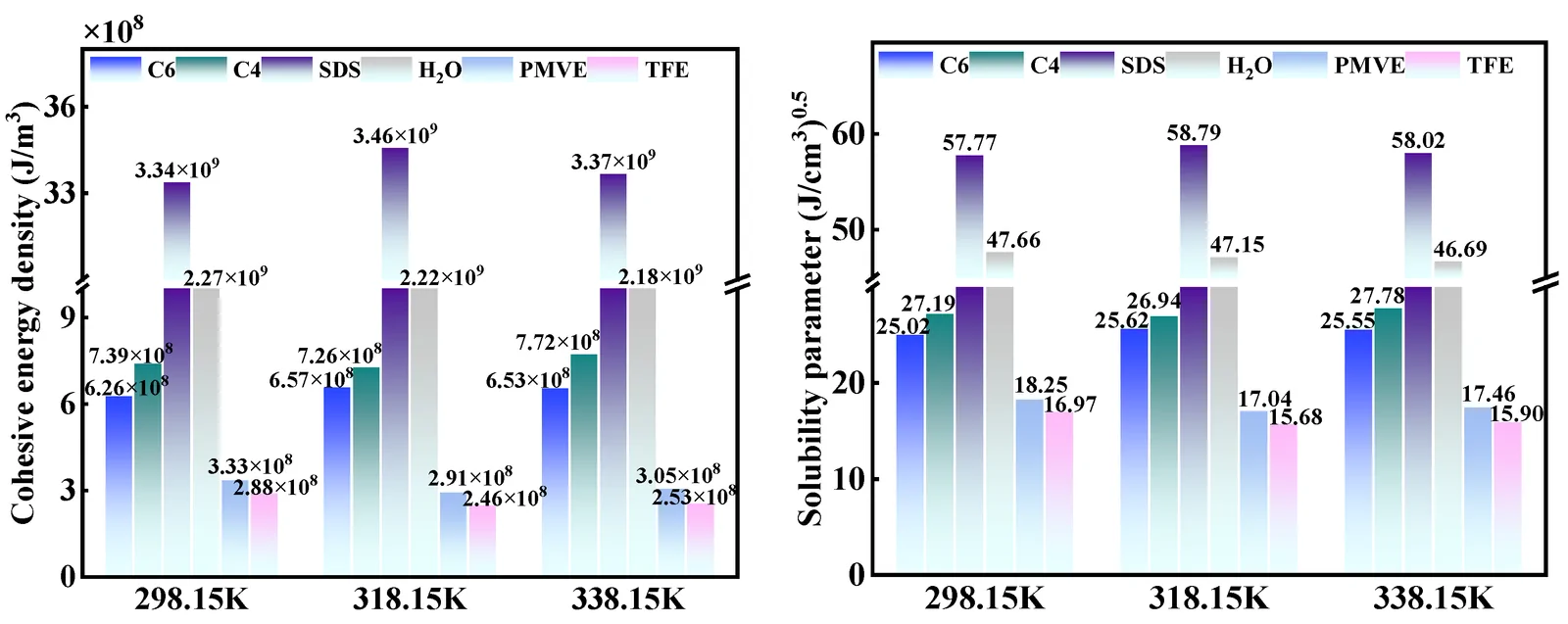
Cohesion energy density and solubility parameters of TFE, PPVE and three surfactants at different temperatures.

To preliminarily elucidate the solubilization mechanism of surfactants with different structures in TFE and PMVE systems, as shown in Figure 2, this study compared the cohesive energy density and solubility parameters of the target monomers and the three surfactants. According to common solvent solubility parameter values, the solubility parameter of H_2_O at 298.15 K is 47.88 (J/cm^3^)^0.5^. The simulated *δ* for H_2_O in this paper is 47.66 (J/cm^3^)^0.5^, with a relative error of only −0.46%, which supports the reliability of the simulation protocol [34]. Both TFE and PMVE exhibit relatively low cohesive energy density and solubility parameters, the physical essence of which stems from their highly fluorinated molecular structures and the low polarizability of the C-F bond. A comparison of the parameter evolution patterns at different temperatures (Table 3) reveals that at 298.15 K, the difference in solubility parameters (Δ*δ*) between the monomers and surfactants is at its minimum, indicating the most favorable thermodynamic conditions for molecular-level dispersion [44]. Among the three surfactants evaluated, the *δ* value of SDS shows a massive discrepancy compared to that of the fluorinated monomers. This high thermodynamic incompatibility suggests that SDS struggles to achieve efficient enrichment of fluorinated monomers. In contrast, the *δ* values of fluorobetaine surfactants are significantly lower than those of SDS and closer to the values of the monomers. This may be because the perfluoroalkyl chains in their structures can significantly enhance affinity with fluorinated monomers through dispersion force matching. Among them, the solubility parameter of C6 is the closest to that of the monomer system. This closer matching may be due to the higher density of perfluorinated groups in the C6 molecular structure; the low polarizability of the fluorine atoms effectively reduces the dispersion force component of the molecule, while its betaine headgroup provides a moderate polar balance, thereby achieving overlap with the fluorinated monomers in the three-dimensional Hansen space [45].

While thermodynamic matching provides a theoretical reference for monomer composition, the actual composition during copolymerization may also be influenced by the differences in reactivity between TFE and PMVE. Therefore, the ideal ratio determined based on solubility parameters should not be directly regarded as a fixed composition for the actual copolymerization process. This study does not aim to optimize feed ratios but rather focuses on the effects of different surfactants on monomer solubilization behavior. Consequently, a 3:1 TFE:PMVE molar ratio was adopted as a unified model composition and maintained consistently across all simulation systems to establish a comparable research baseline. It should be noted that there are currently no reported experimental polymerization kinetic data for the specific TFE/PMVE + PFSC combinations used in this study; thus, this ratio serves only as a model reference for molecular-scale simulations and is not an experimentally verified optimal feed ratio. Within this framework, the simulation results can be used to compare the effects of different surfactants on the spatial distribution and local interactions of fluorinated monomers, providing a molecular-scale reference for understanding solubilization mechanisms and for surfactant screening in subsequent experiments.

### 3.3. Equilibrium Conformations and Spatial Distribution of Solubilization Systems: Equilibrium Snapshots and Relative Concentration Profiles

**Figure 3:**
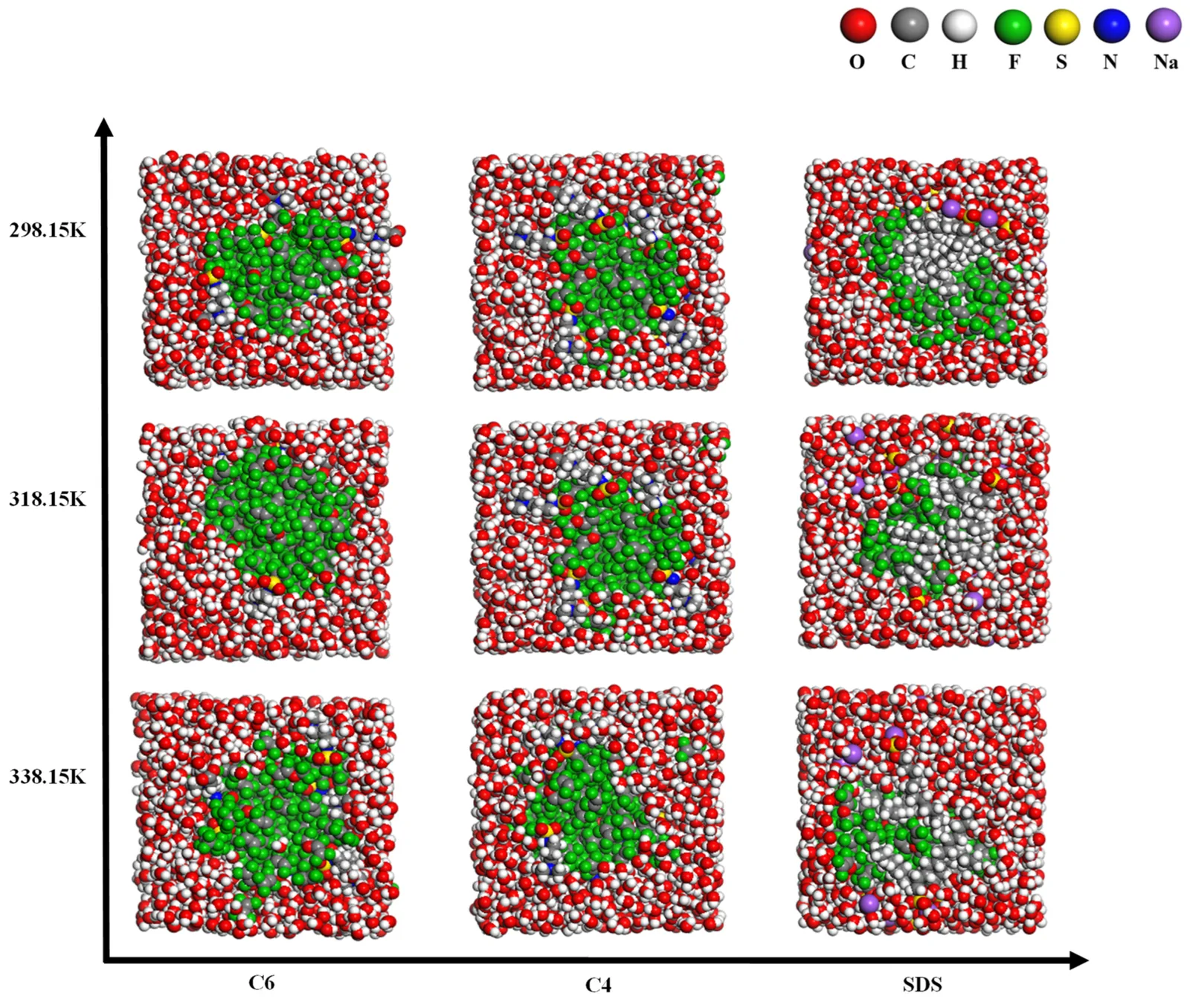
Equilibrium system diagram of the TFE + PMVE + H_2_O + surfactants system at different temperatures.

To intuitively examine the aforementioned predictions based on solubility parameters, this study extracted equilibrium snapshots of each system at the end of the molecular dynamics simulations, as shown in Figure 3. Within the temperature range of 298.15 K to 338.15 K, the fluorinated monomers in the SDS system exhibited a more dispersed spatial distribution compared to the fluorinated systems. This may be attributed to the weaker thermodynamic compatibility between the hydrocarbon tails of SDS and the fluorinated monomers, making it difficult for the monomers to enrich within the surfactant segment regions. In contrast, the fluorinated monomers in the C4 and C6 systems remained primarily distributed in regions rich in perfluoroalkyl chains. At 338.15 K, although the distribution range of monomers in the C6 system expanded and the local density decreased, they generally remained within the fluorocarbon-rich segments; conversely, some fluorinated monomers in the C4 system gradually diffused toward the aqueous phase. As the temperature increased, the fluorinated monomers in the SDS system showed a more pronounced trend of spatial dispersion.

**Figure 4:**
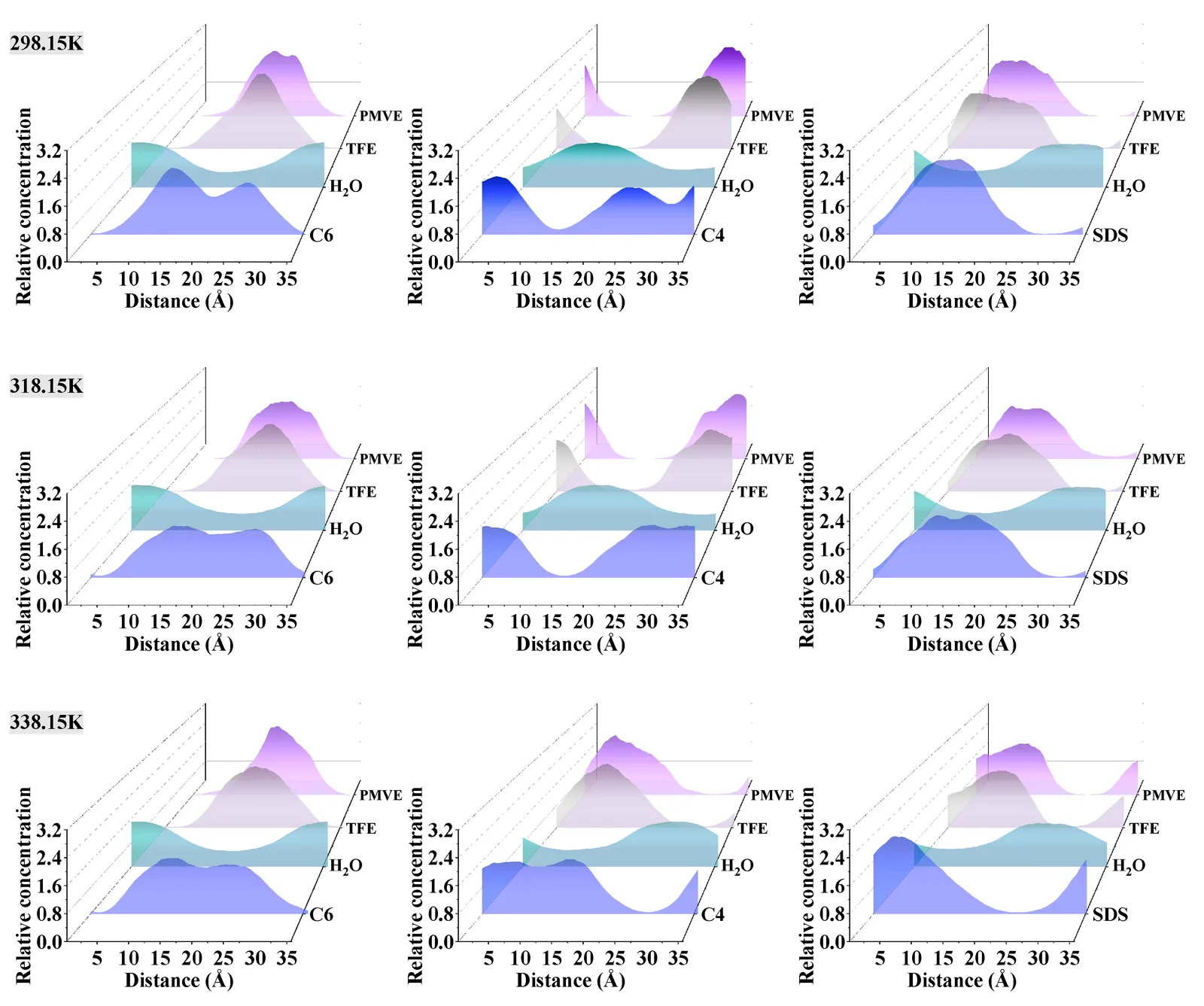
Relative concentration profiles of various components in different TFE + PMVE + H_2_O + surfactant systems at different temperatures.

To further investigate and compare the spatial organization of surfactants and monomers, as well as their behavioral characteristics at the interface, we calculated the relative concentration distribution curves of each component along the *Z*-axis of the simulation box after the system reached equilibrium. As shown in Figure 4, taking the C6 system at 298.15 K as an example, the aqueous phase is enriched at both ends of the box, while PMVE and TFE are concentrated in the center, resulting in a typical three-layer spatial structure. The concentration curves of the surfactant molecules exhibit adsorption spikes at 14 Å and 28 Å, precisely corresponding to the interfacial transition zones where the aqueous phase concentration drops sharply and the organic phase concentration surges, indicating that the surfactants are directionally adsorbed at the water–oil interface to reduce interfacial tension. At the center of the organic phase (approximately 20 Å), the surfactant concentration shows a local minimum, forming a characteristic double-peak distribution at the interfaces with a valley in between, which conformationally reflects the amphiphilic orientation of the surfactants [46]. Additionally, the concentration curves of PMVE and TFE largely overlap and exhibit similar spatial distributions within the fluorine-rich central region, indicating their similar partitioning behavior in this region and suggesting that the perfluoroalkyl chains may promote the local accumulation of fluorinated monomers within the surfactant-rich region [47].

A horizontal comparison of the concentration distribution maps across different surfactant systems reveals that, compared to SDS, the span of the concentration peak bases for fluorinated monomers in the C4 and C6 systems is relatively wider, which is consistent with the schematic diagrams of the equilibrated system configurations. As the temperature increases from 298.15 K to 338.15 K, neither monomer shows an obvious shift toward the aqueous regions at the two sides of the simulation box. Although the temperature rise leads to a slight decrease in the peak concentration values and a broadening of the peak shapes for both monomers and surfactants, the main concentration regions of TFE and PMVE remain largely within the regions occupied by the perfluoroalkyl chains of the surfactants. This characteristic is particularly prominent in the C6 system.

### 3.4. Thermodynamic Driving Force and Spatial Confinement of Microenvironments: Interaction Energy Partition and Microscopic Coordination

Interaction energy reflects the strength of interactions between different molecules. To uncover the thermodynamic driving forces behind solvation, the interaction energies between various components in the equilibrium trajectory were precisely decomposed. The total interaction energy can be calculated using Equation (5) [48]:

*E* = *E_bonds_* + *E_angles_* + *E_inversion_* + *E_cross_* + *E_non-bond_* (5)

where *E_bonds_* represents the bond stretching energy, *E_angles_* represents the bond angle bending energy, *E_inversion_* represents the dihedral angle torsion energy, and *E_cross_* represents the coupling energy. Since no chemical reactions are involved, the bonded terms are not considered. *E_non-bond_* includes electrostatic energy and van der Waals energy, calculated as follows [34]:

(6) $$E_{\mathrm{elec}}=\;\sum_{i,j}\frac{q_{i}q_{j}}{\varepsilon_{0}r_{\mathrm{ij}}}\times \frac{1}{4\pi }$$

(7) $$E_{\mathrm{vdw}}=\sum_{i,j}\varepsilon_{\mathrm{ij}}\left[2{\left(\frac{r_{\mathrm{ij}}^{0}}{r_{\mathrm{ij}}}\right)}^{9}-3{\left(\frac{r_{\mathrm{ij}}^{0}}{r_{\mathrm{ij}}}\right)}^{6}\right]$$

where *E_vdw_* represents the van der Waals energy, and *E_elec_* represents the electrostatic energy; *q_i_* and *q_j_* are the charges of the i-th and j-th ions in the molecule, respectively; *ε* is the potential well depth; and *r_ij_* is the equilibrium distance between the i-th and j-th ions. When *E* is negative, the pairwise interaction between the two atoms manifests as mutual attraction, allowing the system to exist stably [49]. The larger the absolute value of *E*, the stronger the interaction force, the greater the physical affinity and compatibility between components, and the more stable the system becomes.

**Figure 5:**
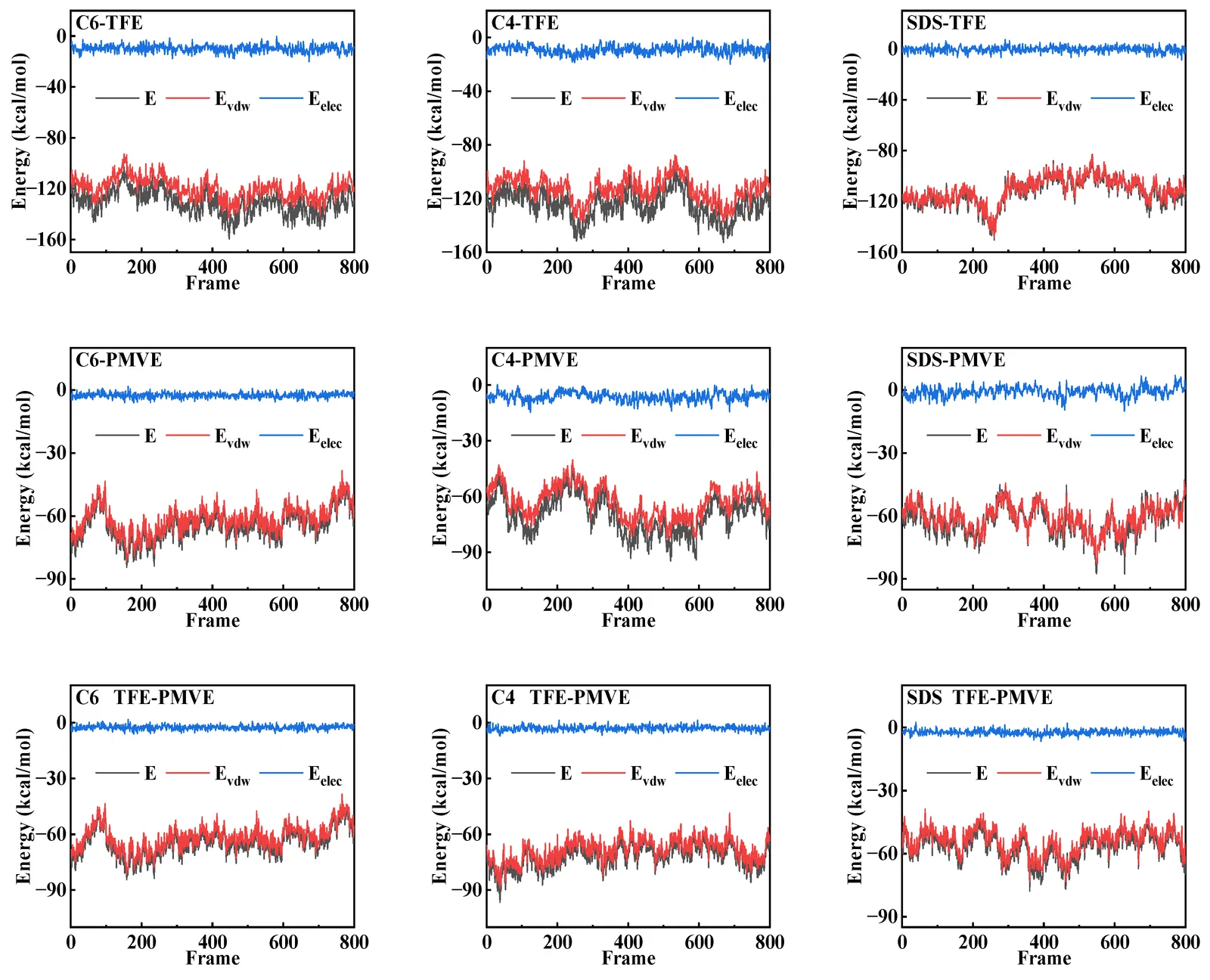
Variation in the interaction energy of different TFE + PMVE + H_2_O + surfactant systems at 298.15 K.

**Figure 6:**
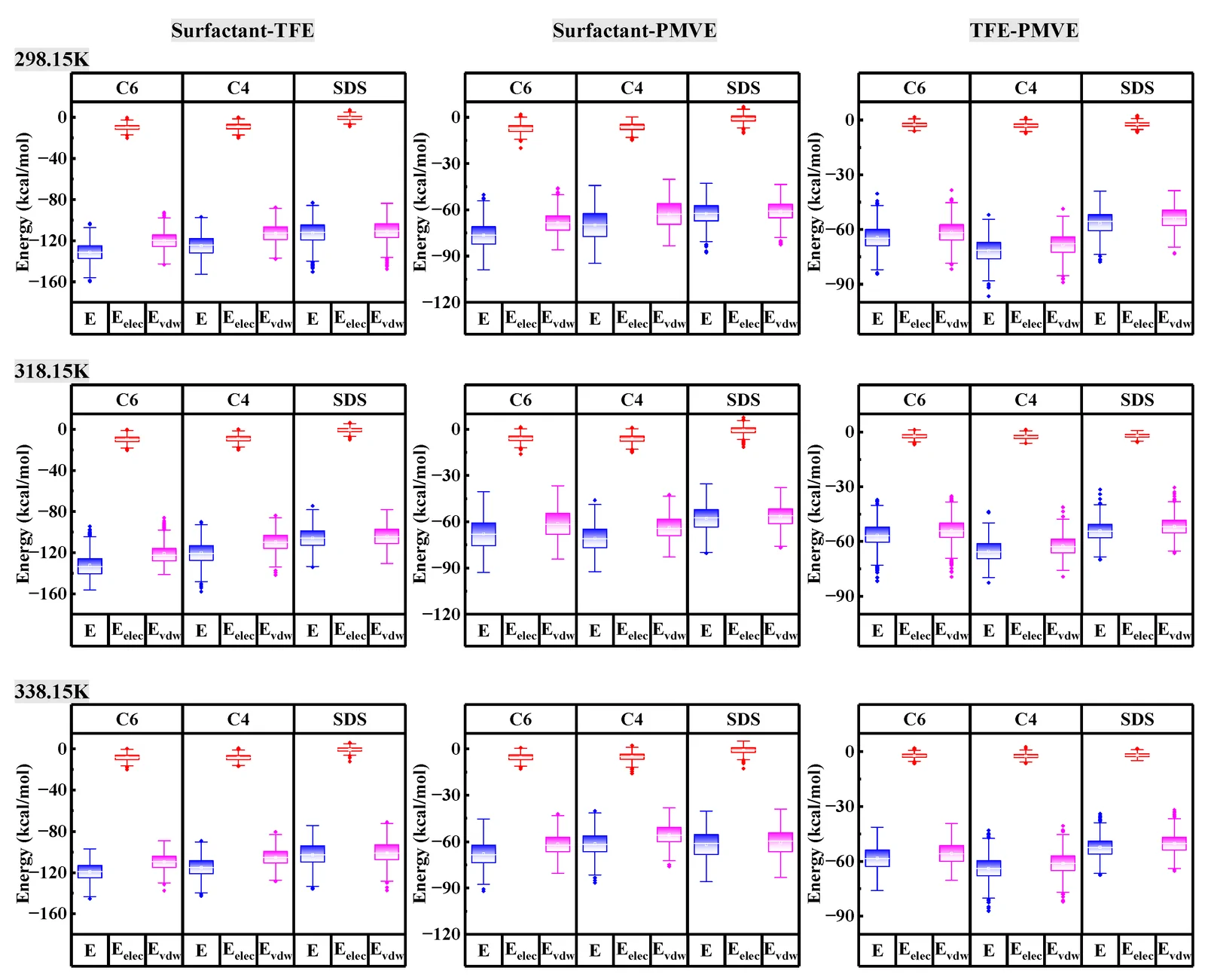
Statistics on interaction energy data of different TFE + PMVE + H_2_O + surfactant systems at different temperatures.

Figure 5 illustrates the evolution of interaction energy for each model as a function of the number of frames at 298.15 K. As shown in the figure, all values are negative, and the electrostatic forces are nearly 0 kcal/mol. The interaction energy data are summarized in Figure 6. In all systems, the absolute value of the van der Waals energy is significantly greater than that of the electrostatic energy, and its distribution highly overlaps with the total interaction energy, indicating that dispersion forces are the dominant contribution to the emulsifier–monomer binding. The absolute values of the interaction energy for fluorinated emulsifiers with monomers are greater than those for SDS, with C6 being the largest. As the temperature increases from 298.15 K to 338.15 K, the absolute values of the interaction energy for all systems show a downward trend, which is attributed to the intensified molecular thermal motion leading to increased intermolecular distances and weakened interactions. Notably, the absolute values of the interaction energy between emulsifiers and monomers (−140 to −160 kcal/mol) are much larger than those between TFE-PMVE monomers (−40 to −80 kcal/mol), indicating that surfactant–monomer interactions make a dominant contribution to the local association of fluorinated monomers.

**Figure 7:**
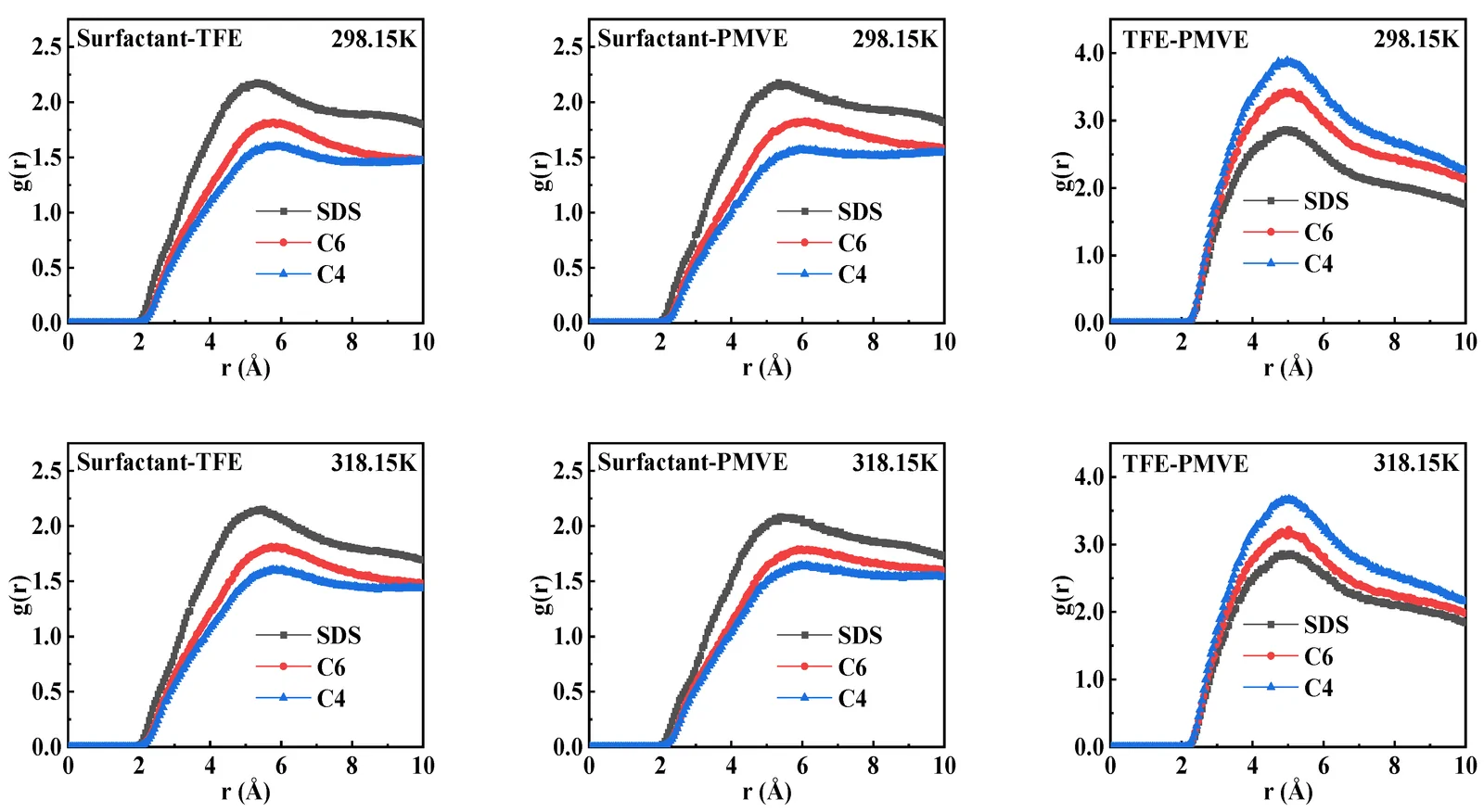

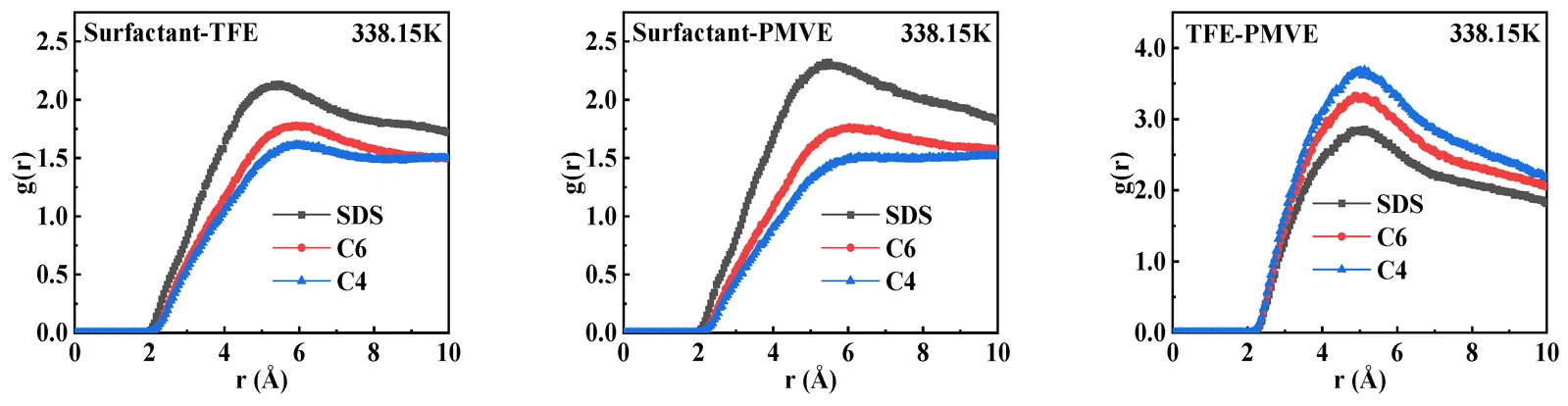
Radial distribution functions of different TFE + PMVE + H_2_O + surfactants at different temperatures.

To quantify the spatial distribution of fluorinated monomers within the surfactant microenvironment and evaluate their interaction strength, this study analyzed the position and distribution of molecules based on the radial distribution function (RDF) (Figure 7). The formula is [50]:

(8) $$g(r)=\frac{\mathrm{dN}}{\rho 4\pi r^{2}\mathrm{dr}}$$

where *N* is the total number of molecules within the distance *r* between the target molecule and the reference molecule, and *ρ* is the system density.

Furthermore, the first-shell coordination numbers between key components were calculated using the following formula [34]:

(9) $$n(r^{\prime })=4\pi \rho \int_{0}^{r^{\prime }}g(r)r^{2}\mathrm{dr}$$

where *n* is the integral value of the radial distribution function from the center point to a specified radius (*r’*), representing the coordination number, which usually refers to the integral within the abscissa range of the valley following the first peak. *r’* denotes the distance to the specified radius on the abscissa, measured in Å; *ρ* is the number density, measured in Å^−3^.

Since statistical properties calculated based on molecular dynamics trajectories may be affected by temporal fluctuations and differences between independent trajectories, this study employs the block averaging method on equilibrated production trajectories to estimate statistical errors. Specifically, the production trajectory is divided into four consecutive and equal-length data blocks, and the statistics for each block are calculated separately to obtain the mean and standard deviation. Furthermore, to evaluate the independence of trajectory sampling, independent replicate trajectories were generated using different random initial velocities and used to compare the consistency of the statistical results.

To further evaluate the impact of trajectory sampling on the calculation of coordination numbers, an independent replicate simulation analysis was performed using the C6–TFE coordination number in the TFE + PMVE + H_2_O + C6 system as an example (Figure S1). The results show a certain degree of fluctuation in coordination numbers between different independent trajectories, reflecting the inevitable statistical uncertainty in finite-time molecular dynamics simulations. However, the average coordination numbers calculated from each independent trajectory are close to the results obtained from the block averaging of the original trajectory, indicating that the original trajectory results possess good statistical consistency. In light of this, subsequent analyses in this paper focus on the overall trend differences between different systems, rather than over-interpreting subtle variations at individual temperature points.

**Figure 8:**
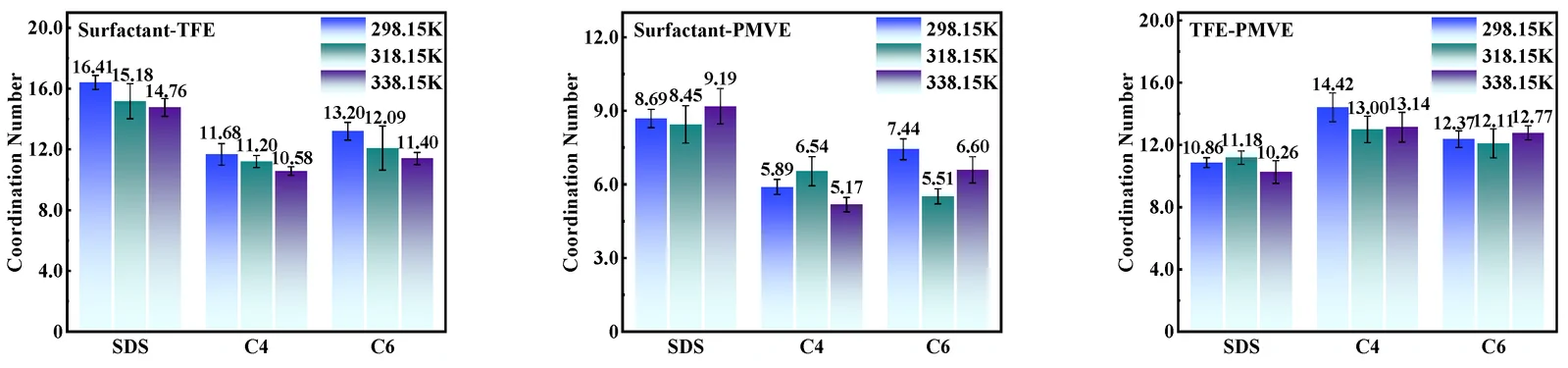
Coordination numbers of different TFE + PMVE + H_2_O + surfactant systems at different temperatures (error bars represent the standard deviation after block averaging).

The calculated coordination numbers between the components in the system are shown in Figure 8. In the traditional hydrocarbon surfactant SDS system, the RDF peak values and coordination numbers between the surfactant and monomers remain relatively high at all temperatures. For instance, at 298.15 K, the coordination numbers for SDS-TFE and SDS-PMVE are 16.41 and 8.69, respectively. Combined with the aforementioned solubility parameter analysis, the thermodynamic matching between SDS and fluorinated monomers is relatively low; therefore, the higher coordination numbers here primarily reflect the local spatial coordination distribution of surfactants and monomers in the SDS system, rather than being directly equated to stronger solubilization.

Further comparing the effects of different perfluorinated chain lengths, the coordination number between the surfactant and the monomer in the C6 system is overall slightly higher than that in the C4 system. Combined with the aforementioned analysis of interaction energy and solubility parameters, this difference may be related to the stronger local interaction between the longer fluorinated segments and the fluorinated monomers, as well as more favorable thermodynamic matching [51].

The spatial correlation between monomers further provides a reference for the spatial confinement effect of fluorinated surfactants. At all tested temperatures, the coordination numbers and RDF peak values between monomers follow the order of C4 > C6 > SDS. The PMVE-TFE coordination numbers in the fluorinated systems (13.00–14.42 for C4 and 12.11–12.77 for C6) are higher than the 10.26–11.18 observed in the SDS system. This indicates that the two monomers possess a more pronounced local spatial correlation within the fluorinated surfactant systems [52].

### 3.5. Local Dynamics and Structural Response Under Thermal Fluctuations: Mean Square Displacement and Radius of Gyration

**Figure 9:**
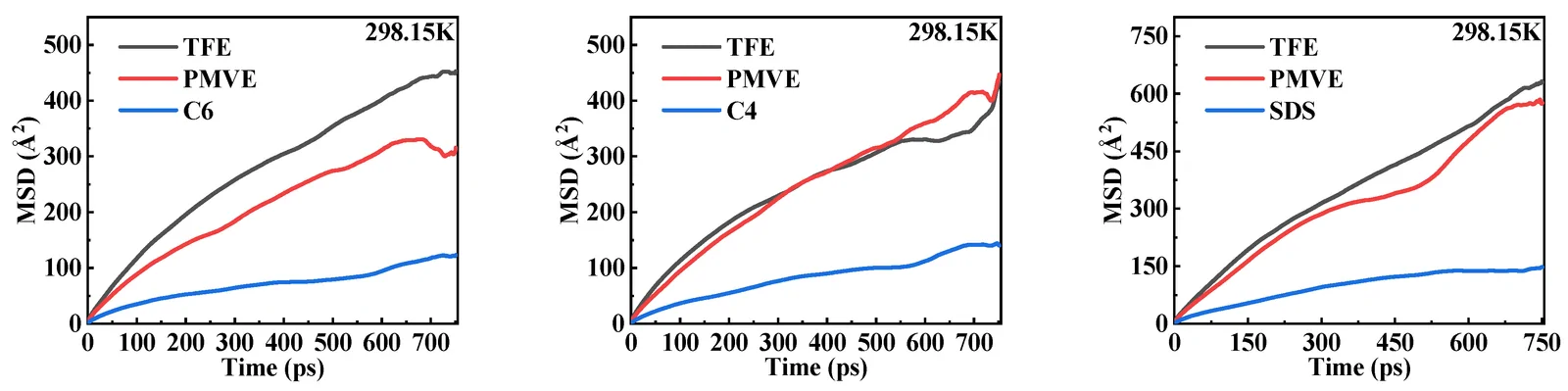

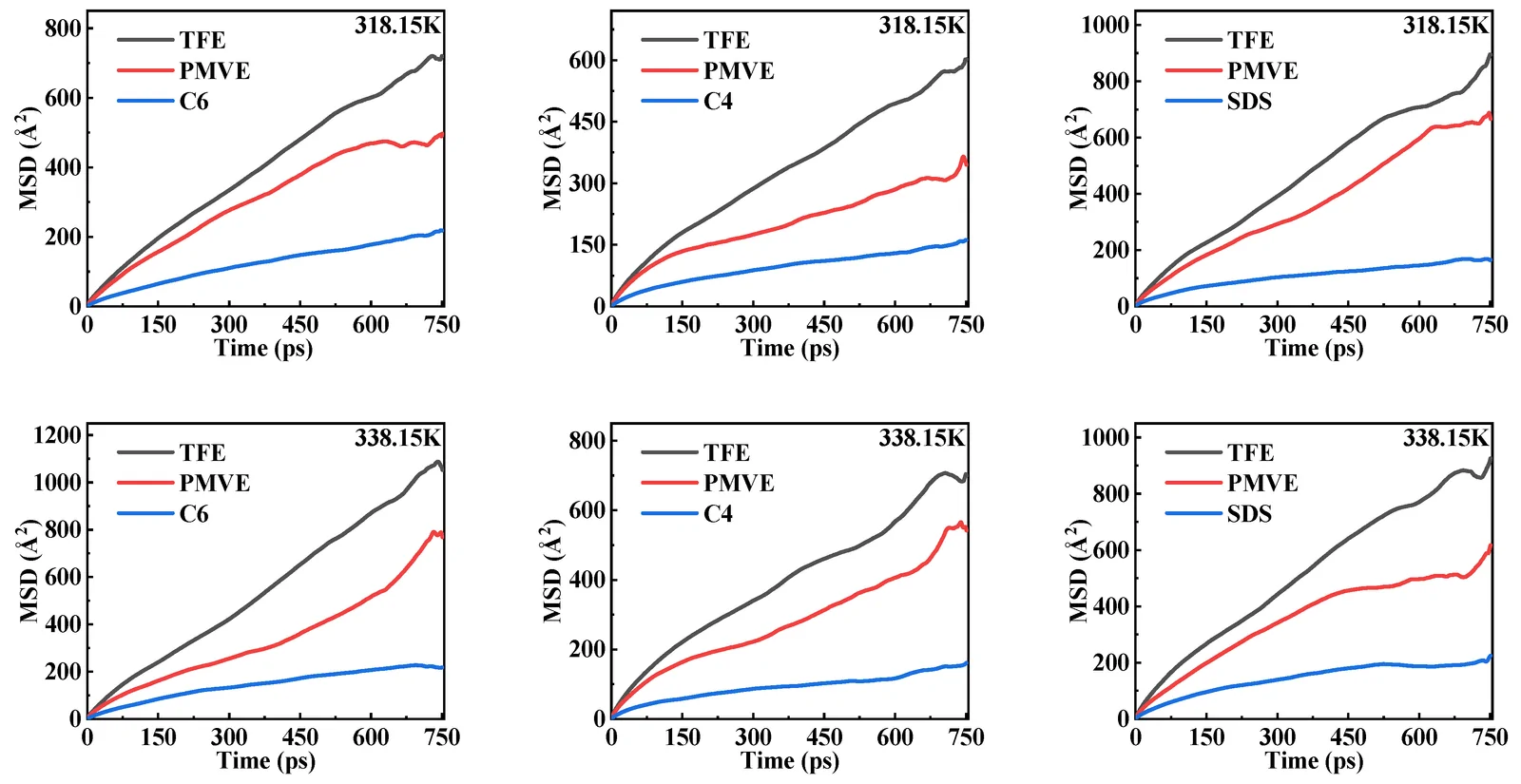
Mean square displacement of various components in different TFE + PMVE + H_2_O + surfactant systems at different temperatures.

To further reveal the stability of the solubilization system under temperature variations, we shifted our research focus to the microscopic motion and diffusion properties of the molecules [53]. The mean square displacement (MSD) of various molecules in different systems was investigated to reflect the degree of deviation in their spatial positions. The MSD increased almost linearly with simulation time (Figure 9), indicating that the system had reached equilibrium [34].

(10) $$\mathrm{MSD}(t)=\frac{1}{N}\sum_{i=1}^{N}|r_{i}\left(t\right)-r_{i}\left(0\right){|}^{2}$$

where *N* is the number of diffusing molecules, *r_i_*(0) is the initial position of molecule *i*, and *r_i_*(t) is the position of molecule i at time *t*.

The diffusion coefficient (D) of the molecules is obtained through the linear relationship between the mean square displacement and the simulation time [54]:

(11) $$D=\frac{1}{6N}\underset{t\to \infty }{\lim}\frac{d}{\mathrm{dt}}\sum_{t=1}^{N}[r_{i}\left(t\right)-r_{i}\left(0\right){]}^{2}$$

where *N* represents the number of diffusing molecules in the system, and the differential term is the slope of the mean square displacement relative to time.

To reduce the impact of trajectory fluctuations on the calculation of diffusion coefficients, this study employs the block averaging method. The entire production trajectory is divided into four equal, consecutive data blocks, and the mean square displacement and corresponding diffusion coefficients are calculated for each block. The final reported diffusion coefficient is the average of the results from these four data blocks, with error bars representing the standard deviation.

**Figure 10:**
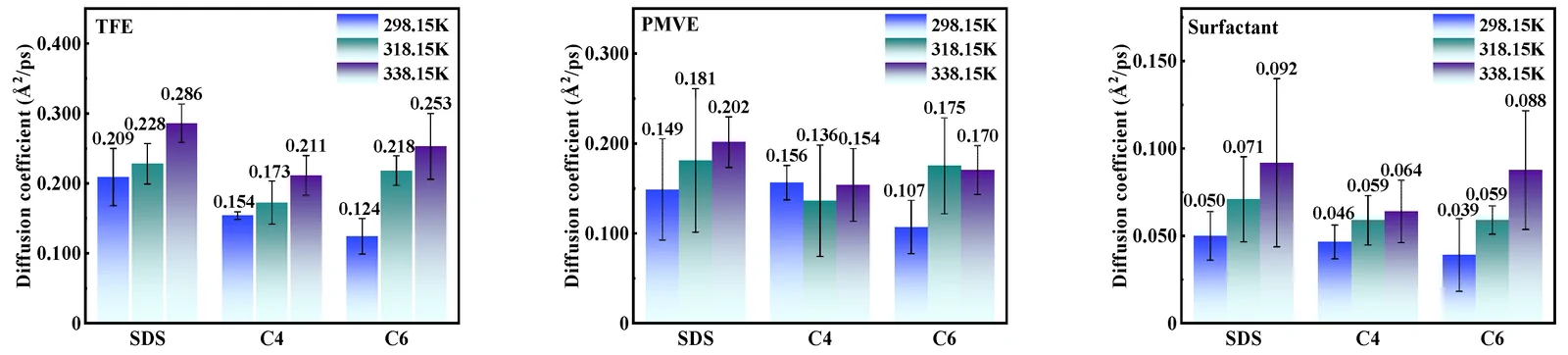
Diffusion coefficient of various components in different TFE + PMVE + H_2_O + surfactant systems at different temperatures (error bars represent the standard deviation after block averaging).

The diffusion coefficients of each species in the system at different temperatures are shown in Figure 10. Overall, the D values of each component exhibit differences related to molecular size and steric hindrance, generally following the order of TFE (0.124–0.286 Å^2^/ps) > PMVE (0.107–0.202 Å^2^/ps) > surfactant (0.039–0.092 Å^2^/ps). Surfactant molecules, characterized by their larger size and subjection to interfacial adsorption and local spatial constraints, exhibit lower overall mobility [55]. In contrast, TFE, with its smaller molecular size, possesses higher translational degrees of freedom and exhibits superior diffusion capability.

Further lateral comparison reveals differences in the dynamic behavior of monomers across different surfactant systems. In the traditional hydrocarbon surfactant SDS system, both TFE and PMVE exhibit high mobility across the entire temperature range. This may be related to the weak thermodynamic matching between the SDS hydrocarbon chains and the fluorinated monomers, resulting in relatively weak local interactions and spatial constraints between the monomers and the surfactant segments. In contrast, the diffusion coefficients of monomers in the C4 and C6 systems are overall lower, indicating that the degree of freedom for monomer migration in fluorinated surfactant systems is relatively small. This kinetic characteristic is consistent with the features of the local fluorine-rich environment derived from the previous concentration distribution and equilibrium structure analyses, further demonstrating that different surfactant systems exert distinct influences on the migration kinetic behavior of monomers.

**Figure 11:**
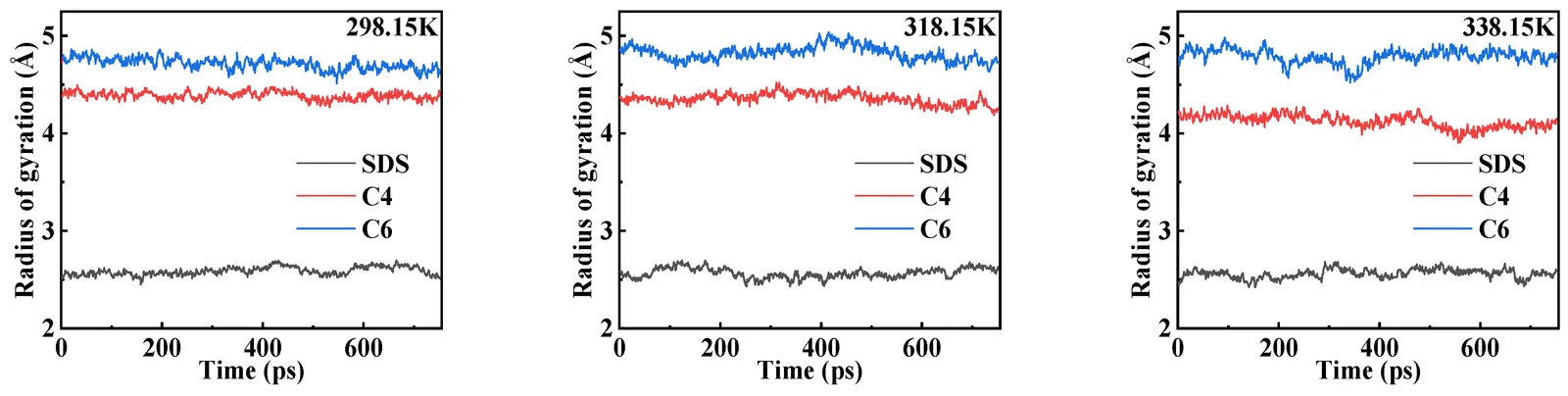
Radius of gyration of surfactant in different TFE + PMVE + H_2_O + surfactant systems at different temperatures.

The radius of gyration (*R_g_*) of the surfactant molecules was further analyzed to evaluate their conformational response to thermal fluctuations. As shown in Figure 11, the three surfactant systems exhibited different conformational sizes, with the overall Rg magnitude remaining in the order of SDS < C4 < C6 across the investigated temperature range. This trend may be related to differences in molecular chain structures and conformational dimensions [56]. As the temperature increased, the Rg fluctuations for all three systems intensified, particularly in the C6 system, reflecting the response of the surfactant chains to thermal perturbations. Combined with the analysis of the system equilibrium phase diagram, the C6 system maintains a relatively stable local spatial organization even under high-temperature conditions. This may be attributed to the greater conformational flexibility of the longer fluorinated segments in C6, which allows it to respond to temperature changes through local conformational adjustments while maintaining the overall spatial organization [57,58].

## 4. Conclusions

This study investigates the solubilization behavior of fluorinated monomers by SDS and PFSC. The surface tension data and water solubility parameter data for PFSC obtained through model simulations are in good agreement with experimental results, supporting the reliability of the calculations. The results indicate that in the SDS system, fluorinated monomers do not exhibit a significant tendency to aggregate toward surfactant-rich regions, which is consistent with the weak thermodynamic compatibility between SDS and fluorinated monomers. In contrast, the PFSC system demonstrates a more ordered spatial organization of fluorinated monomers, with monomers tending to distribute in local regions rich in fluorocarbon chain segments. This aligns with the closer solubility parameter matching between PFSC and fluorinated monomers, as well as interaction energy data dominated by van der Waals dispersion forces. Relative concentration distributions, RDF, and coordination number analyses further support the enhanced local spatial correlation of fluorinated monomers in the PFSC system. Diffusion coefficient results further reveal differences in the migration kinetic behavior of monomers in different surfactant systems; fluorinated monomers in the SDS system exhibit higher diffusion levels, while monomer diffusion in the PFSC system is generally lower. A comparison of perfluorinated chain lengths shows that the C6 system exhibits more favorable thermodynamic compatibility and more stable local spatial organization characteristics with fluorinated monomers, suggesting that longer perfluorinated chains may provide a more stable local environment for fluorinated monomers. Combining the aforementioned structural and kinetic results, the fluorine-rich microenvironment may help maintain the local organization of fluorinated monomers under thermal perturbations.

However, it should be noted that the analysis in this study regarding the local interactions and spatial distribution characteristics between fluorinated monomers and surfactants is primarily based on molecular dynamics simulations. While these results provide interaction information at the molecular scale, the structural features derived from simulations and their correlation with macroscopic solubilization behavior still require further experimental validation. Future research could incorporate dynamic light scattering to characterize the particle size distribution and hydrodynamic radius of the aggregates. Additionally, small-angle neutron scattering or cryo-transmission electron microscopy could provide complementary structural and morphological information. Such experimental studies will help establish the link between the simulated surfactant-rich regions and the aggregation behavior characterized experimentally, and further evaluate their structural response to temperature, thereby enhancing the understanding of solubilization mechanisms and providing guidance for the rational design of environmentally friendly fluorocarbon surfactants.

## Figures and Tables

**Table 1:** Composition of constructed boxes in molecular dynamics simulations.

Number	Composition of System	No. of TFE	No. of PMVE	No. of Surfactants	No. of Water
1	TFE	45	0	0	0
2	PMVE	0	15	0	0
3	SDS	0	0	15	0
4	C6	0	0	15	0
5	C4	0	0	15	0
6	H_2_O	0	0	0	1000
7	TFE + PMVE + SDS + H_2_O	45	15	15	1000
8	TFE + PMVE + C4 + H_2_O	45	15	15	1000
9	TFE + PMVE + C6 + H_2_O	45	15	15	1000

**Table 2:** The surface tension of C4 and C6.

Surfactant	*γ_exp_* (mN/m)	*γ_sim_* (mN/m)	Relative Error (%)
C6	12 [30]	10.37	−13.58
C4	15 [30]	13.62	−9.20

**Table 3:** Differences in solubility parameters between various surfactants and monomers at different temperatures.

Temperature (K)	Surfactant	Δ*δ*_PMVE_	Δ*δ*_TFE_
298.15	C6	6.8	8.0
	C4	9.0	10.2
	SDS	39.6	40.8
318.15	C6	8.6	9.9
	C4	9.9	11.2
	SDS	41.8	43.1
338.15	C6	8.1	9.7
	C4	10.3	11.9
	SDS	40.5	42.1

## Data Availability

The raw data supporting the conclusions of this article will be made available by the authors on request.

## Supplementary Materials

The following supporting information can be downloaded at: https://www.mdpi.com/article/10.3390/polym18172056/s1. Figure S1: Assessment of independent trajectory sampling on the coordination number calculation of C6–TFE in the TFE + PMVE + H_2_O + C6 system at 318.15 K. The analysis includes the original trajectory and three independent replicate trajectories. The averaged coordination number was calculated from all four trajectories. Error bars for individual trajectories were obtained by block averaging, while the error bar of the overall average represents the standard deviation among the four trajectories.
