# Supplementary material for: Solubilization Mechanism of Eco-Friendly Fluorocarbon Surfactants for Fluorinated Monomers: Perfluorinated Chain Length Effect and Dynamic Thermal Response
Source: Polymers (Basel). 2026 Aug 24;18(17):2056. doi: 10.3390/polym18172056 (PMC13567937; doi:10.3390/polym18172056)
Supplement: Supplementary file 1 [file polymers-18-02056-s001.zip › polymers-4484276-supplementary.pdf]

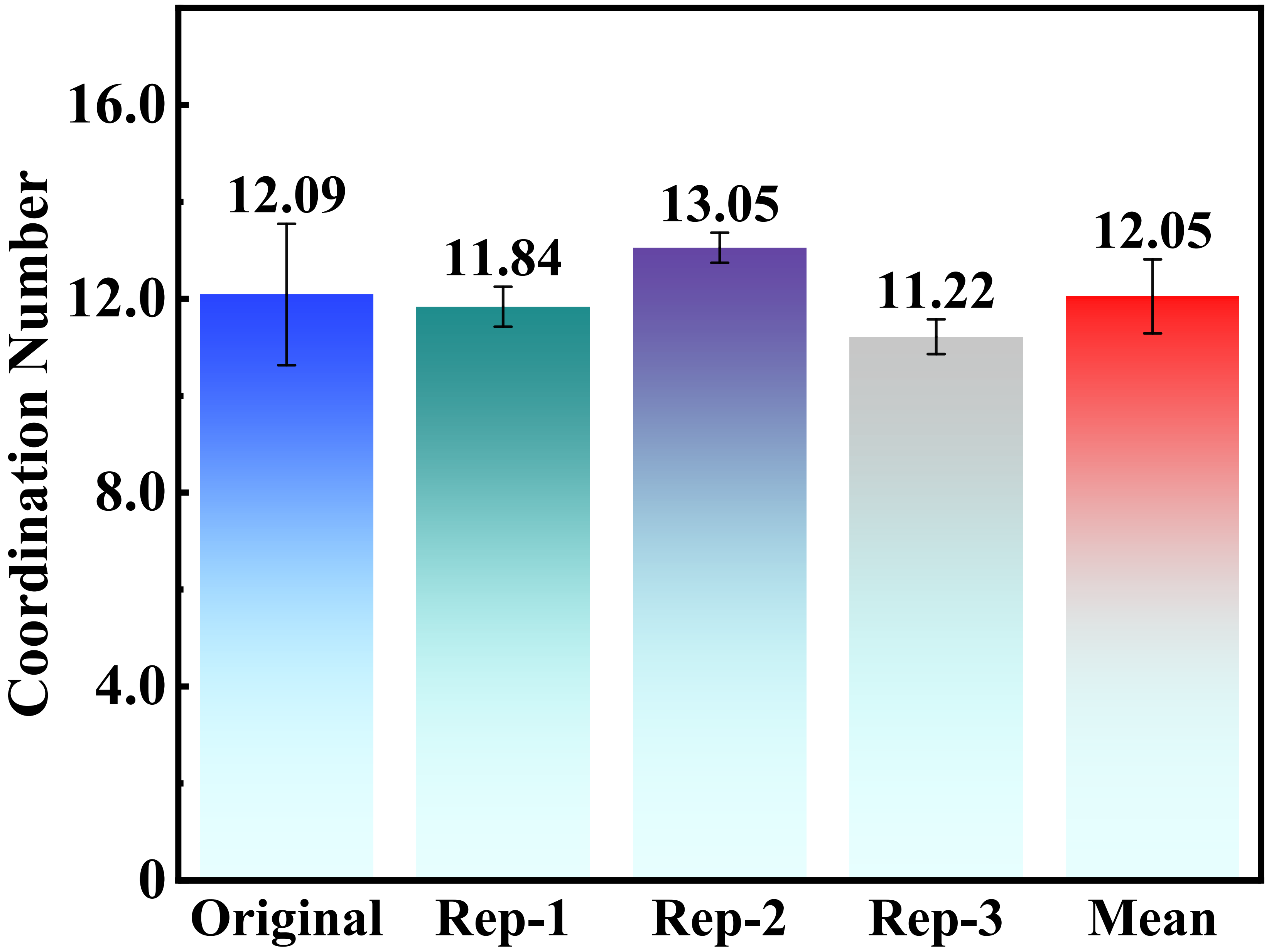

Fig. S1. Assessment of independent trajectory sampling on the coordination number calculation of C6–TFE in the TFE + PMVE + H_2O + C6 system at 318.15 K. The analysis includes the original trajectory and three independent replicate trajectories. The averaged coordination number was calculated from all four trajectories. Error bars for individual trajectories were obtained by block averaging, while the error bar of the overall average represents the standard deviation among the four trajectories.
